# State of the art and challenges in sequence based T-cell epitope prediction

**DOI:** 10.1186/1745-7580-6-S2-S3

**Published:** 2010-11-03

**Authors:** Claus Lundegaard, Ilka Hoof, Ole Lund, Morten Nielsen

**Affiliations:** 1 The Technical University of Denmark - DTU, Dept. of Systems Biology, Center for Biological Sequence Analysis - CBS, Kemitorvet 208, DK-2800 Kgs. Lyngby, Denmark; 2 Utrecht University, Theoretical Biology/Bioinformatics, Padualaan 8, 3584 CH Utrecht, The Netherlands

## Abstract

Sequence based T-cell epitope predictions have improved immensely in the last decade. From predictions of peptide binding to major histocompatibility complex molecules with moderate accuracy, limited allele coverage, and no good estimates of the other events in the antigen-processing pathway, the field has evolved significantly. Methods have now been developed that produce highly accurate binding predictions for many alleles and integrate both proteasomal cleavage and transport events. Moreover have so-called pan-specific methods been developed, which allow for prediction of peptide binding to MHC alleles characterized by limited or no peptide binding data. Most of the developed methods are publicly available, and have proven to be very useful as a shortcut in epitope discovery. Here, we will go through some of the history of sequence-based predictions of helper as well as cytotoxic T cell epitopes. We will focus on some of the most accurate methods and their basic background.

## Challenges from infectious diseases

From the dawn of life, there has been a constant risk of infection by foreign organisms so that only host organisms that have developed an effective protection against these pathogens survived through evolution. On the other hand, this has put an evolutionary pressure on the pathogenic organisms to circumvent the developed protection mechanisms. Especially single-celled organisms and viruses, which generally have a relatively short generation time occasionally combined with a high mutation rate, have succeeded in finding loopholes in the protection. This million-year old arms race has led to the development of a defense system, the immune system, which itself consists of genetically diverse unicellular components that can evolve within the host organism when put under selective pressure. Occasionally, pathogens have evolved that efficiently could infect a specific host organism leading to high mortality. This is typically seen after a change of host [1]. Obviously, too high mortality among the host species would logically also lead to the pathogenic organism's own end. Due to geographic and biological barriers, such disasters generally hit only locally and were limited to neighboring populations, while physically isolated populations avoided infection [2]. Today there are no longer any major restrictions on mobility and contact between human populations, which increases the small but present risk of a new pathogen posing a threat for the existing civilization. Several examples from the past few years have further exposed such threats; the SARS outbreak in 2003 did relatively quickly spread to several continents [3], and a high mortality has been observed in cases where certain strains of the avian flu, Influenza A H5N1 infect humans [4]. The recent Influenza A H1N1 pandemic, originating from pigs, is the latest example of how extensive these infections can be [5,6]. Fortunately, humans have recently been spared from the emergence of new pathogens that are at the same time both very contagious and extremely deadly. Chronic infections, which have little acute mortality but moderate to high mortality in longer terms are another growing problem. Examples of such are infections with hepatitis C virus (HCV), human immunodeficiency virus (HIV), and tuberculosis (TB). 

## The immune system and vaccines

The most effective protection against infections is through vaccination. Most vaccines today exist as an inactivated or more harmless form of the pathogenic organism. In several cases, there are problems with either the efficacy, side effects, or that the pathogen is constantly changing and thus escapes the vaccine's protection. The latter issue is one of the major obstacles to, for instance, a long lasting Influenza A vaccine. Vaccines take advantage of the features of the adaptive immune system. The immune system in general reacts to foreign substances and organisms when discovered in the body. The innate immune system gives a fast and unspecific response, which does not change with repeated occurrences of the same pathogen. The innate immune response might eliminate the intruder by itself but it also signals to the adaptive part of the immune system [7]. An existing effective humoral immunity is an extremely potent way of preventing an actual infection as the intruder will be eliminated immediately. For this reason vaccine development has traditionally been focusing on developing effective antibody responses, which can be obtained using totally inactivated pathogens, parts thereof, or even single proteins in case of vaccines against toxins such as tetanus or diphtheria [8]. However, to obtain strong and long lasting memory it appears that a strong T cell response is often needed [9]. The cellular arm of the immune system consists of two parts; cytotoxic T lymphocytes (CTL), and helper T lymphocytes (HTLs). Both CTL and HTL recognize peptides that are presented on the cell surface to the immune cells by the major histocompatibility complex (MHC) molecule, which in humans is referred to as the Human Leucocyte Antigen (HLA). While HTLs are needed for B cell activation and proliferation to produce antibodies against a given antigen, CTLs perform surveillance of the host cells and recognize and kill infected or malfunctioning cells that present non-self peptides (epitopes) [10]. In a vaccine context, the relevant proteins expressed by a given pathogen are the proteins that will be determining for a good immune response, i.e., the antigens. The part of the antigen that is recognized by the immune system is the epitope, and in the case of both the CTL and the HTL such epitopes consist of small, 8-20 amino acid long polypeptides. 

## CTL epitopes

In the MHC class I pathway, peptides from endogenous antigens bound to class I MHCs are presented to CTLs, which are carrying the CD8 receptor (CD8+ T cells). To be presented, a precursor peptide is usually first generated by the proteasome, a large cytosomal protease complex [11,12]. For further processing, the peptides must enter the endoplasmic reticulum (ER). This generally happens by active transport mediated by the transporter associated with antigen processing (TAP) [13]. However, some peptides can enter the ER even with an absent TAP function, as some presented peptides originate from proteins containing a signal peptide. These proteins may enter the ER through the Sec61 transporter complex [14] and should be considered especially when dealing with infected or malignant cells that might have an impaired TAP function [15,16]. This is highly relevant for peptides binding to MHCs belonging to the abundant A2 HLA serotype where TAP independent presentation is responsible for up to 10% of the A2 restricted epitopes [17]. During or after transport into the ER a potential epitope must bind to the MHC class I molecule [18,19] generally facilitated by the helper protein tapasin [20,21], before it can finally be presented on the cell surface. The most selective step in the classical MHC class I pathway is binding of a peptide to the MHC molecule. To be an epitope, i.e., to raise a CTL response, a peptide should generally bind with an affinity stronger than 500 nM [22]. As a support for this general assumption, Moutaftsi et al. [23] found that of the 49 epitopes that are responsible for 95% of the total CD8+ T cell response against a vaccinia challenge in mice 90% bind MHC with an affinity stronger than 500 nM. The work by Moutaftsi et al. also clearly underlines the usefulness of predictions in vaccine development, as only a very limited subset of peptides derived from the vaccinia proteome had to be tested to identify epitopes responsible for 95% of the CTL response. The tested subset included only the best 1% predicted of all the possible peptides.

## MHC class I binding predictions

Since MHC binding of a peptide is a necessary requirement for its recognition by a T cell, predicting their capability to bind MHC molecules can facilitate and significantly cost-reduce the identification of T cell epitopes in a set of peptides. The majority of peptides binding to MHC class I molecules have a length of 8–11 amino acids, even though several longer epitopes have been identified [24]. The second position and the C-terminal position of the peptide are typically the most important for binding, and these positions are referred to as anchor positions [25,26]. For some alleles, the binding motifs further have auxiliary anchor positions. For example, peptides binding to the human HLA-A*0101 allele have position 3 as an additional anchor [25,27,28]. Only few different amino acids are tolerated at the anchor positions of peptides binding to a given MHC allele. The discovery of such allele-specific motifs led to the development of the first algorithms for prediction of peptide binding [29-31], which essentially determined whether a peptide did or did not match the binding ‘motif’ of the MHC molecule.

As more data has accumulated, it has become possible to go beyond the match/mismatch classification of a motif prediction. By use of statistical methods, scores can be calculated for each possible amino acid at each position in a peptide, leading to an Lx20 scoring matrix where L is the length of the peptide. For predictions, it is then assumed that the amino acids at each position along the peptide sequence contribute a certain binding energy (the score from the matrix), which can independently be added up to yield the overall binding energy of the peptide [32-34]. This type of approach is used by the EpiMatrix (commercial) [35], BIMAS (http://www-bimas.cit.nih.gov/molbio/hla_bind/) [33], SYFPEITHI (http://www.syfpeithi.de/) [36], RANKPEP (http://bio.dfci.harvard.edu/RANKPEP/) [37], Gibbs sampler (http://www.cbs.dtu.dk/biotools/EasyGibbs/) [38], SMM (http://tools.immuneepitope.org/analyze/html/mhc_binding.html) [39], and ARB (http://tools.immuneepitope.org/analyze/html/mhc_binding.html) methods [40]. These methods differ in the way they derive the matrix coefficients. Some are trained by statistical methods that analyze how often a given amino acid is seen at a given position in binding versus non-binding peptides. Matrix coefficients can also be determined by a machine learning procedure, which aims at finding the coefficients that best explain the observed binding data. This can be done by interpreting the matrix scores for a peptide as predicted binding affinities and by minimizing the distance between predicted and measured values. This is the approach utilized by SMM, which is presently the best performing matrix method in published benchmark studies [41,42].

 Matrix-based methods cannot take correlated effects into account, i.e., where the contribution to the binding affinity of an amino acid at one position depends on amino acids at other positions in the peptide. Higher order methods like artificial neural networks (ANNs) and Support Vector Machines (SVMs) are ideally suited to take such correlations into account [43-48]. These methods can be trained with data either in the format of binder/non-binder classification, e.g. binders from the SYFPEITHI database of eluted peptides [36], or as real affinity data as can be found in the Immune Epitope Database (IEDB) [49,50]. Likewise the predictors can either be trained to output a score that correlates with the chance that a given peptide is a binder or to output a score that corresponds to a predicted affinity [51]. The ANN based predictor *NetMHC *[45,47,52] was trained using both sequence input from affinity data mainly found in the IEDB as well as output from matrices generated by SYFPEITHI [36] eluted peptides using the Gibbs sampler approach [47]. In two recent benchmark comparisons the *NetMHC-3.0* implementation was the most successful method including higher order sequence correlations [41,42]. The *NetMHC* method has been further improved in the *NetMHC-3.2* version (http://www.cbs.dtu.dk/services/NetMHC) by training on data with larger peptide and allelic coverage. (Lundegaard et al., J. Imm. Meth., submitted). As mentioned earlier, most epitopes and MHC binding peptides discovered to date are of length 8, 9, or 10 amino acid residues, even though longer epitopes have been identified, mostly hendecamers, but also a few even longer [24]. Data driven prediction-algorithms for MHC class I binding are for the most part limited to predict the same lengths as they have been trained on, and in the IEDB, very few examples of such longer peptides exist today. Of all unique eluted MHC binding peptides in the current version of the IEDB database, only 10% are longer than 10 residues and 4% are longer than 11. Some MHC:peptide binding methods have been developed using the information of the three dimensional structure of known complexes. These methods should in principle be able to predict binding also of longer peptides. However, not even on nonamer peptides are these methods as accurate as the data driven methods [53,54], and have to our knowledge not been benchmarked on longer peptides. It has been shown, though, that predictions from methods trained on nonamer peptides can be used to predict the affinity of longer peptides, which has been benchmarked with peptides of a length up to 11 residues [55]. This system has been implemented into the *NetMHC* method. To summarize: of the prediction methods publicly available online, the neural network based *NetMHC* performs best on the tested evaluation sets, followed by the matrix based *SMM *[41,42]. The *SMM* training and prediction code is freely available [39]. The implementation of online consensus MHC class I prediction tools is currently in progress at the IEDB site (Björn Peters, personal communication), as an approach of combining different prediction methods might give even better results [56]. How accurate the best of methods are can be exemplified by comparing the prediction accuracy of the single methods with the correlation between different experimental methods [42]. Both the *SMM* and the *NetMHC* methods are available via the IEDB website (http://www.immuneepitope.org) [56], and *NetMHC* is additionally accessible from http://www.cbs.dtu.dk/services/NetMHC. 

**Figure 1:**
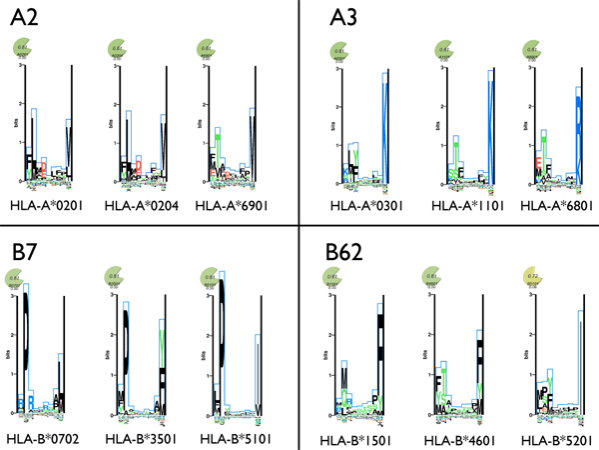
**Depiction of the supertype concept.** Example alleles including alleles common in the western European populations were assigned to four supertypes using the scheme from Sidney et al. [63]. The amino acid preferences at each position in a nonamer peptide is shown for each of the alleles using sequence logo plots taken from MHCMotifViewer [129]. Amino acids with positive influence on the binding are plotted on the positive y-axis, and amino acids with a negative influence on binding are plotted on the negative y-axis. The height of each amino acid is given by their relative contribution to the binding specificity.

 Today more than 2000 HLA alleles have been identified, and as they in principle bind different peptide repertoires, the task of mapping the peptide preferences for each and every one of these would be experimentally overwhelming. Initially only the most common alleles were examined, but it was soon clear that some alleles were sharing peptide preferences often, which did not always correlate with the amino acid sequence similarity of the compared alleles [57]. This discovery lead to the concept of supertypes, where several alleles are clustered into groups (supertypes), based on the degree of functional similarity (Figure 1) [57-63]. In this approach still only the most common alleles were studied, however, the population coverage of identified epitopes could be theoretically extrapolated assuming complete peptide binding overlap between alleles within a given supertype.

**Figure 2:**
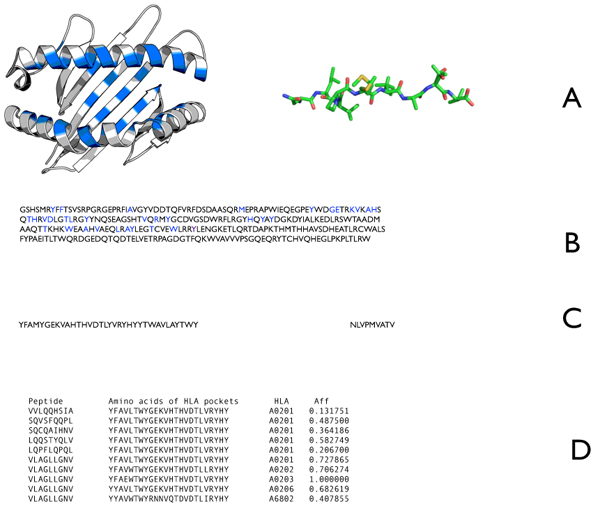
**Description of the NetMHCpan approach.** A) Amino acids used for prediction are residues from the MHC alpha chain that are found to be in contact with the peptide using structural data (blue in left) and the full binding peptide (right). B) The identified MHC residues in the amino acid sequence of HLA-A*0201 are labeled blue. C) The labeled residues from B presented as a pseudo sequence (left) and the peptide sequence (right). D) Pairs of peptide sequences (left) and pseudo sequences (second from left) are presented to the ANN with the experimentally determined log scaled affinity (far right). The displayed allele information is not an input to the ANN. During the training the weights are adjusted in order to minimize the error between predicted output and the assigned affinity.

 Lately, the amount of publicly available binding data has increased significantly mainly due to the huge effort funded by NIH resulting in the IEDB database [49]. This database is now very extensive both in terms of the number of different peptides and the number of different MHC alleles for which binding data exist. Furthermore, the MHC class I binding data are very homogeneous in quality as more than 99% of the quantitative binding data in the IEDB database generated since 2006 were generated by two comparable assays developed in the laboratories of A. Sette and S. Buus [64-67]. More than 95% of the class I data has been generated since 2005, and less than 2% before 2001. However, besides leading to MHC prediction systems, which now cover a large number of different HLA alleles [42,68], this large growth in the amount of MHC peptide-binding data has enabled the development of new so-called pan-specific algorithms. These pan-specific methods go beyond the conventional single allele approach and are able to predict peptide-binding to HLA alleles, for which the sequence is known but only limited or no experimental binding data are available [68-73]. The architecture of the training system of *NetMHCpan* has been outlined in a way that takes both the peptide sequence and the MHC contact environment into account (Figure 2). Polymorphic positions in the MHC assumed to be in contact with a residue in a bound peptide have been mapped in order to extract a pseudo sequence representing the given MHC molecule [72]. This pseudo sequence is used as input in the training coupled with a peptide sequence and the measured affinity of the given peptide:MHC. Thus, the machine learning method behind the predictions is trained to be able to combine the information provided by the MHC sequence and the peptide sequence in order to predict a specific binding affinity. In this way, the system can combine information from the MHC sequence with the peptide sequence to derive cross correlations and is able to predict the outcome of MHC:peptide combinations that it has not encountered during the training. Several pan-HLA methods have been evaluated in a large-scale benchmark, and the outcome of this evaluation demonstrated the power of the pan-specific methods. Not only do these methods predict peptide-binding affinities to previously uncharacterized MHC molecules but the incorporated training setup also boosts the predictive performance for already characterized alleles by leveraging information from neighboring MHC molecules [74], see Table 1. *Kiss *[70] is available from http://cbio.ensmp.fr/kiss/, *ADT *[71] is available at http://atom.research.microsoft.com/hlabinding/, and *NetMHCpan *[72,73] is available at http://www.cbs.dtu.dk/services/NetMHCpan. The latter server has implemented the approach of extrapolating from 9mers to prediction of binding for peptides up to 11 residues in length [55] and allows prediction for all known HLA-A, -B, and -C alleles, as well as some non-human primate, mouse and pig MHC alleles.

**Table 1:** Performance of available pan-specific predictors.

Performance Measure	***Kiss***	***ADT***	***NetMHC***	***NetMHCpan***
Pearson CC	0.455	0.488	0.593	0.620
Spearmans Rank CC	0.44	0.522	0.561	0.600
AUC	0.734	0.756	0.807	0.820

Performance values taken from [74]. The prediction servers have been evaluated on a set of binders to 17 HLA-A alleles and 16 HLA-B alleles. The data had not been used for training of any of the tested alleles.

 In an attempt to perform a completely unbiased benchmark of different MHC binding prediction approaches, several groups have participated in a competition that has been held in connection with the ICANN 09 conference (http://www.kios.org.cy/ICANN09/MLI.html). The binding to the MHC alleles HLA-A*0101, HLA-A*0201, and HLA-B*0702 were to be predicted for a total of 177 10mer peptides and 265 9mers. The results of this competition placed *NetMHC-3.2* and *NetMHCpan-2.2* as the best performing methods on the benchmark set, and a prediction approach using the simple mean of the predictions from these two methods was awarded the first price among the 20 competing methods (Vladimir Brusic, personal communication, submitted to J. Imm. Meth.).

## Prediction of other MHC class I pathway events

In the following, we describe predictions of proteasomal cleavage and TAP binding. The proteins responsible for these events are basically monomorphic, and developers of prediction methods do not face the same allele problem as is present for MHC binding prediction. This should in principle make the task of developing accurate prediction methods easier. This is, however, not the case as the assays determining the cleavage and binding are not developed for high throughput to the same extent as is the case for MHC:peptide binding assays. For this reason data for these two processing events are in general scarce.

 The complex enzymatic specificity of the proteasome makes the prediction of its cleavage patterns highly challenging. The proteasome comprises multiple catalytically active sites, each with a distinct specificity [75,76]. A further complication is that two versions of the proteasome exist. The proteasome that functions in most cells and which has the main task of recycling superfluous or malfunctioning proteins is constitutively expressed and is therefore called the constitutive proteasome. An inducible version of the proteasome, the immunoproteasome, is expressed when a cell receives signals from the innate or the adaptive immune system indicating that it should enter an ‘alarm’ state. The immunoproteasome has catalytic subunits with different specificity than the constitutive proteasome. This change gives rise to a catalytic complex, which cleaves proteins into fragments that are better processed by the other players in the MHC class I pathway [77]. The outcome of proteasomal cleavage has been considered in two separate ways when it comes to predictive purposes. One way is to predict the chance of a given position in the protein sequence to be cleaved. Another approach is to predict the likelihood that a given peptide fragment will arise after proteasomal cleavage. *FragPredict*, which is publicly available as a part of *MAPPP* service (http://www.mpiib-berlin.mpg.de/MAPPP/), takes the latter approach and consists of two sequential algorithms. The first algorithm uses a statistical analysis of cleavage-enhancing and -inhibiting amino acid motifs to predict potential proteasomal cleavage sites [78]. The second algorithm predicts the likelihood that a given peptide fragment will arise using the results of the first algorithm as an input. The second algorithm has been developed to select the most likely fragments to be generated. The model calculates the time-dependent degradation based on a kinetic model of the 20S proteasome [79]. The *PAProC* (http://www.paproc.de) method predicts in vitro proteasomal cleavages performed by human and wild type and mutant yeast proteasomes. The influence of different amino acids at different positions is determined by using a stochastic hill-climbing algorithm [80] based on the experimentally verified in vitro cleavage and non-cleavage sites [81]. A weight matrix method has been developed which predicts both constitutive- and immunoproteasomal cleavage specificity [82] trained on the very limited in vitro proteasomal digest data available. The NetChop [83] method has been trained using information from C termini of naturally processed MHC class I ligands. No other significant endopeptidases or exopeptidases processing the C-terminus of peptides have been observed in the cell compartments involved in the class I pathway. Therefore the C-termini of MHC I presented peptides are believed to be created by proteasomal cleavage. Since some of these ligands are generated by the immunoproteasome and some by the constitutive proteasome, such a method should predict the combined specificity of both forms of proteasomes. *NetChop-2.0* was evaluated to be the best-performing predictor on an independent evaluation set [84]. The SVM based *Pcleavage* proteasomal cleavage predictor, which is available online, has a published performance comparable to that of NetChop-2.0 [85]. An update of the NetChop method to version 3.0 [77] consists of a combination of several ANNs, each trained using a different sequence-encoding scheme of the data. NetChop-3.0 (http://www.cbs.dtu.dk/services/NetChop) has an increased sensitivity as compared to NetChop-2.0, without lowering the specificity. A method using SVM predictions and apparently achieving very good results has recently been published [86]. In their evaluation, however, the developers do not compare AROC/AUC values, described by [87], which is the best suitable value for comparison of the performance of these kind of predictors [53]. The method is not available as software, code, or server, and still awaits independent evaluation. Finally, a new method predicting the likelihood that a given peptide originates from proteasomal cleavage has been implemented as a publicly available server (http://peptibase.cs.biu.ac.il/PepCleave_II/) [88], and according to the published evaluation this method works well. Good benchmarks for a comparison of the usefulness of the different types of predictions have not yet been implemented.

 Relatively few methods have been developed to predict the specificity of TAP. Daniel et al. [89] have developed ANNs using 9-mer peptides, for which the TAP affinity was determined experimentally. Surprisingly, they found that some MHC alleles such as alleles belonging to the HLA-A*02 family have some natural ligands with very low TAP affinities. This could either be because TAP ligands can be trimmed in the ER before binding to MHC molecules [90] and that a TAP ligand therefore often enters the ER as a precursor to the MHC binding peptide, or it could be due to alternative entrance routes, as described earlier. Peters et al. [91] used an SMM based matrix to predict TAP affinity for peptides of length 9 or longer.They used this model to show that natural A2 ligands are well transported by TAP in form of precursor peptides, hence confirming the trimming hypothesis by Fruci et al. A number of different TAP binding prediction methods have since been published as recently reviewed [54,92]. Several methods utilizing machine-learning algorithms have been published with a predictive performance superior to the SMM method. It must be mentioned, though, that these methods probably suffered from overtraining, and only a single SVM based method, *TAPREG* (http://imed.med.ucm.es), appears to have been able to match the predictive performance of the SMM based method using a new benchmark dataset [93]. However, while *TAPREG* works only for nonamer peptide predictions, the SMM based method was further generalized to work on peptides that are longer than 9 amino acids. It was found that mainly the three N-terminal residues and the C-terminal residue had influence on the binding affinity of TAP binding peptides [91,93]. Thus, the affinity of peptides longer than 9 amino acid residues can be predicted by using matrix scores only for the three N terminal residues and the C terminal residue in the peptide.

 The action of ERAAP peptidase has also been shown to be important for peptide binding [24,94], and the importance of tapasin in the class I presentation pathway has recently become evident [20,21,95,96]. Data regarding these players are still very scarce and their function has not been examined in relation to epitope prediction, but it is likely that methods for prediction of these events will be developed as data become available.

## Integrated CTL epitope predictions and optimal population coverage

Although predictions of MHC binding in itself can be used to rank the possible CTL epitopes quite accurately [97,98], even better predictions should be attainable if other steps in the antigen processing and presentation pathway were modeled and included in a final prediction. Several attempts have been made to predict the outcome of two or more steps involved in antigen processing and presentation: *MAPP* (http://www.mpiib-berlin.mpg.de/MAPPP/) [99], *NetCTL* (http://www.cbs.dtu.dk/services/NetCTL) [100], *NetCTLpan* (http://www.cbs.dtu.dk/services/NetCTLpan), *MHCpathway* (http://tools-int-01.liai.org/analyze/html/mhc_processing.html) [101], *EpiJen* (http://www.darrenflower.info/EpiJen/) [102], and *WAPP* (http://www-bs.informatik.uni-tuebingen.de/Services/WAPP) [103]. All of these methods attempt to predict antigen presentation by integrating peptide:MHC binding predictions with one or more of the other events involved in the antigen presentation pathway. How well do these methods perform, and which of the methods work best? In a benchmark, a set of verified epitopes can be used as the positive data set. But having only positive data, it is only possible to get a sensitivity score, and methods that will predict any peptide as an epitope will reach the highest rank. On the other hand, a negative data set (containing peptides that cannot induce an immune response) is difficult to define because it is impossible to guarantee that a peptide will never be an epitope in any individual expressing a given HLA allele. To circumvent this problem, epitopes from extensively studied pathogens, such as HIV, are often used as the positive set, and all other peptides that are present in the whole proteome of the same pathogen and have never been shown to give an immune response are chosen as the negative set (non-epitopes), thus assuming that they will at least have a very low probability of being epitopes. A comparison has been published calculating the predictive performance of several publicly available MHC-I presentation prediction methods [104]. The outcome, using such a large-scale benchmark on known HIV epitopes (http://www.cbs.dtu.dk/suppl/immunology/CTL-1.2/HIV_dataset) revealed that the *NetCTL* and *MHCpathway* methods were ranked the most accurate with >75% of the epitopes ranking among the top 5% peptides sorted by the prediction score [104]. The majority of the described methods only work for a limited number of MHC alleles. To date only the *NetCTLpan* method has integrated the described pan-specific MHC binding prediction systems with predictions of proteasomal cleavage and TAP translocation [98]. 

 When testing predicted epitopes for response in patients or donors the success rate is around 10% depending on selected cutoff and pathogen [23,105,106]. Since the affinity predictions are far more accurate than this there might be other issues to address. These could be inherent issues such as stability of the peptide:MHC complex (half life), the influence of tapasin on successful MHC loading, MHC competition, or holes in the T cell repertoire. But also the fact that many pathogens interfere with the players in the classical MHC class I pathway might influence the epitope repertoire [15]. The outline and outcome of a selected set of epitope discovery experiments have recently been reviewed elsewhere [51].

## Helper T cell epitopes

Helper T cells with a T cell receptor (TCR) specific for antigen-derived peptides must be activated to get strong B cell responses [107]. The epitope recognized by a helper TCR is usually somehow connected to the epitope that is recognized by the B cell receptor, but the two different receptors do not necessarily recognize overlapping epitopes or even epitopes from the same protein. T cells can recognize internal peptides that do not need to be a part of the surface-surface interactions with the B cell receptor. HTLs, which normally carry the CD4 receptor and are therefore also called CD4+ T cells, recognize peptides presented by the MHC class II molecule on the surface of professional antigen presenting cells such as macrophages, dendritic cells, and B lymphocytes. Peptides presented by class II MHCs usually originate from internalized proteins, thus, class II peptide presentation follows a different path than the MHC class I presentation pathway [108]. In short, MHC class II molecules associate with the invariant chain (Ii) in the ER and the MHC:Ii complexes accumulate in endosomal compartments. Here, Ii is degraded, while another MHC-like molecule which in humans is called HLA-DM, loads the MHC class II molecules with the best available ligands originating from endocytosed antigens that have been degraded in the lysosomes partly simultaneously with the MHC maturation process. The peptide:MHC class II complexes are subsequently transported to the cell surface for presentation. 

 In contrast to MHC class I, peptide affinity data for MHC class II have been generated using a diverse set of experimental assays by a large number of different groups. About 80% of the quantitative data has been produced using one single assay type, whereas 20 groups using more than five different assay types produced the remaining 20%. Less than 80% of the data were produced after 2006, and more than 15% of the data were produced before 2001 [109]. Most binding data describing the specificity of MHC molecules are equilibrium binding affinity values. Binding affinity might not be the only relevant feature for the characterization of epitopes. Binding stability might be equally relevant because the avidity of the MHC peptide complex to bind T cells clearly depends both on the equilibrium binding constant and the stability of the complex. Complementing the MHC binding data with peptide stability measurements may, thus, lead to improved epitope predictions. As a result of the open ends of the MHC class II binding cleft, peptides may bind in multiple registers. Several conflicting studies have shown both positive and negative effects of including such multiple binding registers into the prediction of MHC class II binding, and no consensus has been reached in the field as to how big the effect of multiple binding registers would be for an accurate description of the binding specificity. Finally, for naturally processed MHC ligands and CD4 epitopes, factors other than peptide–MHC binding can influence the peptide immunogenicity, including susceptibility to proteolytic activity in the endosome/lysosome and peptide/antigen abundance in the antigen-presenting cell.

## MHC class II binding predictions

**Figure 3:**
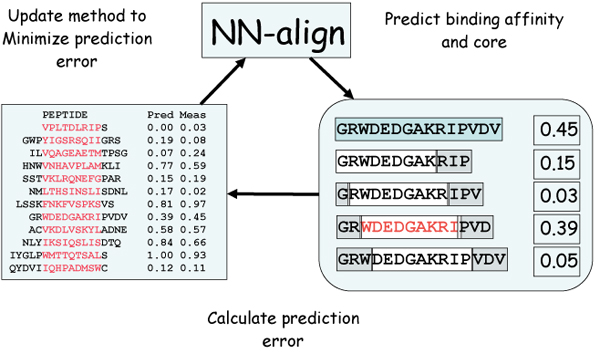
**Schematic overview of the NN-align algorithm.** The artificial neural network (initially with assigned random weights) is used to predict the binding affinity for a given peptide (right panel). The peptide (shown in light blue) is partitioned into overlapping 9mers, and the binding affinity is predicted encoding the 9mer binding core combined with information about the peptide flanking residues (PFR), the length of the PFR and the peptide length as described in the text. The binding affinity of the peptide is assigned from the highest scoring sub-peptide (shown in red). Next, the ANN weight configuration is updated using back-propagation to minimize the squared error between the predicted and measured binding affinities. This is repeated in a cycle for all peptides in the training data set for a given number of iterations.

Unlike MHC class I molecules, the binding cleft of MHC class II molecules is open-ended [110], which allows for the bound peptide to have significant protrusions at both ends. As a result MHC class II binding peptides have a broader length distribution typically of eleven to twenty residues [111]. However, the majority of the binding interaction is mediated by a 9 amino acid residue core sequence of the bound peptide. This complicates binding predictions, as the identification of the correct alignment of the binding core is a crucial part of identifying the MHC class II binding motif [38,56]. Identifying this core is difficult, as the MHC class II binding motifs have relatively weak and often degenerate sequence signals. The majority of MHC class II binding prediction methods are based on the assumption that the peptide–MHC binding affinity is determined solely from a nine amino acid binding core motif. An early effort, *TEPITOPE* developed by Jürgen Hammer [112], used this assumption. The data were obtained by phage display and binding to a selected set of HLA-DRB1 molecules with a changing central 9mer core of the presented peptide. Position specific scoring matrices (PSSM) were derived using statistical analysis of the amino acids observed at each position in binding versus non-binding peptide cores. Such PSSMs were generated for a number of selected HLA-DRB1 alleles and, using structurally derived data, the anchor positions in the peptides were associated with certain binding pockets in the MHC molecule. Assuming that these binding pockets were mutually independent, virtual PSSMs for HLA-DRB1 alleles, for which no data was available, were created by matching amino acid pocket residues of the uncharacterized allele to pockets for the alleles with characterized binding motif. For a long time, this method was the best method for MHC class II binding prediction. Since *TEPITOPE* was originally only made available for PC use, the PSSMs were later derived from publications and made publicly available as a part of the web accessible class II predictor *ProPred* (http://www.imtech.res.in/raghava/propred/) [113]. Even though binding data became available for naturally processed peptides, e.g., from SYFPEITHI [36] it proved difficult to make prediction systems that significantly exceed the accuracy of *TEPITOPE*/*ProPred*. One of the major obstacles has been the identification of the 9mer binding core within these generally longer peptides. Several attempts have been made using more sophisticated methods such as Gibbs sampling [38] or SVMs [114]. The assumption that binding can be predicted from a 9mer core alone is clearly an oversimplification as it is known that peptide flanking residues (PFR) on both sides of the binding core may contribute to the binding affinity and stability of the peptide:MHC complex [115]. Some methods for MHC class II binding have attempted to include PFRs indirectly, in terms of the peptide length, in the affinity prediction [116]. Later, it was demonstrated that including PFRs in MHC class II predictions does in fact improve the prediction accuracy [117]. The method SMM-align (http://www.cbs.dtu.dk/services/NetMHCII-1.1), which implements this approach, has been shown to perform best by independently conducted validations [56,118]. Most of the methods for MHC class II binding predictions have been trained and evaluated on very limited data sets covering only a single or a few different MHC class II alleles, making it very difficult to compare the different performance values and establish generality of the methods. A recent large-scale comparison of prediction methods for MHC class II binding [56] covered 14 HLA-DR (human MHC) and two mouse class II alleles. Recently, an ANN-based method, NN-align (http://www.cbs.dtu.dk/services/NetMHCII-2.2), has been published [119] as an extension to the *SMM-align* method. As depicted in Figure 3, the *NN-align* method uses the current weights optimized in the previous training round to select the optimal 9mer core and PFRs for each of the peptides within the training set. The ANN weights are then optimized on the basis of the errors between the predicted binding affinity using the newly defined core and PRFs. Now, the cores and PFRs are in turn redefined based on the new weights and the iteration is continued until the error ceases to decrease on an external part of the training set not used to optimize the weights. This method works significantly better than previously published methods, but awaits external independent evaluation. Besides the previously described 14 HLA-DR alleles, the updated *NetMHCII-2.2* method includes prediction for six of the most common HLA-DQ and DP alleles. 

 In a way the TEPITOPE approach was already an early pan-specific predictor, but class II predictions have now further benefited from the increasing number of data points available, both regarding the number of peptides and alleles. Pan-specific predictors have been developed covering all HLA-DRB alleles [120] and as the amount of data increases this trend will likely proceed to the other class II loci, DQ and DP.

Even though significant improvements have been made on MHC class II:peptide binding predictions, we are still far from the accuracy obtained in class I predictions. Regarding the usefulness of class II predictions, the lower accuracy is to some extent compensated for by the fact that longer peptides can be used (containing a higher number of possible epitopes each) and that class II MHCs are more promiscuous.

A number of epitope discovery experiments have been performed were MHC class II binding predictions, mainly *TEPITOPE*/*ProPred*, have been included as a filtering step [109,121]. As another example of a recent Th1 epitope discovery effort where MHC class II binding predictions have been integrated is the work of S.A. Mustafa [122]. Here, it was shown that MPT63, a major secreted protein of Mycobacterium tuberculosis, induced moderate Th1 cell reactivity. Analysis of MPT63 hosted peptides for binding to 51 HLA-DR alleles, using ProPred, showed that MPT63 sequences could bind to all the 51 alleles, and nine of the ten peptides of MPT63 were predicted to bind promiscuously. 

## Selection of an optimal epitope pool

Searching for potential T cell epitopes can be guided using *in silico* screening procedures as explained above. Genome wide screening procedures will often identify thousands of potential epitope candidates caused by genomic diversity of the pathogen and the HLA allelic diversity of a given host population. Due to economic and practical limitations, only a small set of epitope candidates can be handled in subsequent epitope validation assays. Several methods have been published recently aiming at identifying a peptide subset that will provide optimal pathogen genomic and HLA coverage in a given population [105,123-125]. The method by Fischer et al. aims at designing mosaic protein with maximal 9-mer peptide coverage of the pathogen genomic diversity. The EpiSelect method described by Perez et al. [105] aims at identifying sets of CTL epitopes with maximum coverage of the genomic variation of the pathogen. All available variants of an organism of interest are screened for peptides predicted to bind to a given allele or supertype representative. The peptide-binder predicted to be present in most of the variable pathogenic strains is selected first. In repetitive selection rounds, new predicted binders are selected according to a scheme that maximizes the overall coverage of the pathogenic strains and leaves as few strains as possible uncovered. This algorithm thus goes one step further than the method by Fischer et al. [123], and includes the HLA restriction in the peptide selection. In the published study, epitopes were predicted for allele representatives of 9 supertypes using NetCTL. For each of the supertypes, peptides were consecutively selected by the EpiSelect scheme. Of 184 peptides tested against blood monocytes from 31 HIV patients infected with various HIV subtypes 114 (62%) were recognized by at least one study subject, and 45 were novel epitopes. Using the EpiSelect algorithm, Perez et al. [105] were able to demonstrate how it is possible to detect and evaluate both the magnitude and breadth of epitope-specific CTL responses in a genetically diverse population infected with different HIV subtypes using a very limited set of HLA class I supertype-restricted epitopes, thus demonstrating the high power of these methods. An alternative approach was taken in the work by Toussaint et al. [125] where a set of peptides with maximum likelihood of eliciting a broad and potent immune response was selected from a user-defined set of predicted or experimentally determined epitopes covering different HLA alleles and pathogen genomic variants.

## Conclusions

Almost two decades ago, MHC peptide-binding data were available for only a few human and mouse alleles. Even from this scarce amount of data, it was found that prediction of new potential epitopes could be performed with a decent accuracy. The large polymorphism of the MHC genomic region and especially of the MHC genes themselves became more and more clear. This challenged the usefulness of identified epitopes as vaccines since many epitopes would be needed to cover a reasonable part of a given population, which would require tremendous resources to be invested in the experimental validation of the predicted epitopes. For more than a decade, the supertype concept has been a highly valuable tool for limiting the number of epitopes needed in an epitope-based vaccine with broad population coverage. However, recent studies have demonstrated that supertypes do provide a strong oversimplification of the peptide binding diversity of the different MHC molecules, and that different MHC alleles within a given supertype often will restrict very different peptide repertoires [126,127]. To entail a detailed understanding of which T cell epitopes can be restricted by a given host, it has therefore become apparent that full HLA typing is required in combination with the recent advances in pan-specific MHC class I binding predictions. Several large scale studies have demonstrated that based on such detailed information, the vast majority of positive T cell responses can be explained [128,129]. These studies also underline that supertype associations may lead to poor or even wrong interpretations of the observed immune correlates. 

Despite the great advances in the accuracy and allelic coverage of methods for prediction of peptide binding to MHC molecules, a great proportion of recent papers published on the subject of rational T cell epitope discovery apply relatively ancient methods like *BIMAS* and *SYPHITHI* for MHC class I and *TEPITOPE*/*ProPred* for MHC class II [109]. This is surprising because many benchmark studies have shown that state-of-the-art data-driven methods significantly outperform these older methods also when it comes to identification of MHC ligands and T cell epitopes. 

It is apparent that at present MHC:peptide binding in silico models can significantly enhance the outcome of epitope discovery experiments. However, there is no doubt that human interpretation by experienced immunologists is necessary in order to correctly interpret and validate the outcome of such prediction systems. Today the most fruitful work seems to be done in collaborations between experimentalists and bioinformaticians. 

The CTL epitope prediction algorithms are today at a level of accuracy where they have already been proven useful in high throughput and full genome based epitope discovery. This gives hope that the methods themselves can be used as analytic tools for investigations of systems biology nature e.g., host/pathogen interactions, and simulate the development of the immune system under specific stimuli. We do strongly believe that in the near future the number of MHC class II binding data will increase significantly, which will lead to the development of new predictive methods and will enhance the performance of existing methods. Furthermore, ongoing experiments indicate, that class II predictions, even at the current level, can be of significant help in Th epitope discovery efforts (Annika Karlsson, personal communication).

## List of abbreviations

HCV: hepatitis C virus; HIV: human immunodeficiency virus; TB: tuberculosis; CTL: cytotoxic T lymphocytes; HTL: helper T lymphocytes; MHC: major histocompatibility complex; HLA: Human Leucocyte Antigen; ER: endoplasmic reticulum; TAP: transporter associated with antigen processing; ANN: artificial neural networks; SVM: support vector machine; IEDB: immune epitope database; TCR: T cell receptor; PSSM: position specific scoring matrix; PFR: peptide flanking region.

## Competing interests

Individuals of the author group have within the last five years been funded by the European Commission (LSHBCT-2003-503231, LSHB-CT-2004-012175) and the US National Institutes of Health (HHSNN26600400006C, HHSN266200400025C, HHSN266200400083C, HHSN272200900045C). No other financial or non-financial competing interests to be declared.

## Authors’ contributions

CL wrote the initial draft and MN, OL, and IH contributed to the development and improvement of the manuscript by rewriting paragraphs and writing significant additions to the text. All authors read and approved the final manuscript.
